# 2-Meth­oxy­carbonyl­pyridinium tetra­chlorido(pyridine-2-carboxyl­ato-κ^2^ *N*,*O*)stannate(IV)

**DOI:** 10.1107/S1600536811001930

**Published:** 2011-01-22

**Authors:** Ezzatollah Najafi, Mostafa M. Amini, Seik Weng Ng

**Affiliations:** a Department of Chemistry, General Campus, Shahid Beheshti University, Tehran 1983963113, Iran; b Department of Chemistry, University of Malaya, 50603 Kuala Lumpur, Malaysia

## Abstract

In the reaction of pyridine-2-carb­oxy­lic acid and stannic chloride in methanol, one equivalent of the carb­oxy­lic acid is protonated at the amino site and is also esterified, yielding the title salt, (C_7_H_8_NO_2_)[SnCl_4_(C_6_H_4_NO_2_)]. The Sn^IV^ atom in the anion is *N*,*O*-chelated by a pyridine-2-carboxyl­ate in a *cis*-SnNOCl_4_ octa­hedral geometry. The cation is linked to the anion by an N—H⋯O hydrogen bond.

## Related literature

For a related organotin structure, see: Nowell *et al.* (1983 ▶).
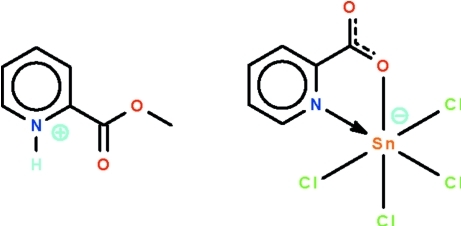


## Experimental

### 

#### Crystal data

(C_7_H_8_NO_2_)[SnCl_4_(C_6_H_4_NO_2_)]
*M* *_r_* = 520.74
Orthorhombic, 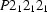

*a* = 8.8898 (3) Å
*b* = 10.3571 (3) Å
*c* = 20.0938 (7) Å
*V* = 1850.09 (10) Å^3^
*Z* = 4
Mo *K*α radiation
μ = 1.98 mm^−1^
*T* = 100 K
0.30 × 0.25 × 0.20 mm

#### Data collection

Agilent SuperNova Dual diffractometer with an Atlas detector
Absorption correction: multi-scan (*CrysAlis PRO*; Agilent Technologies, 2010 ▶) *T*_min_ = 0.588, *T*_max_ = 0.693
6191 measured reflections
3787 independent reflections
3679 reflections with *I* > 2σ(*I*)
*R*_int_ = 0.021

#### Refinement

*R*[*F* ^2^ > 2σ(*F* ^2^)] = 0.021
*wR*(*F* ^2^) = 0.049
*S* = 0.96
3787 reflections
222 parameters
1 restraint
H atoms treated by a mixture of independent and constrained refinement
Δρ_max_ = 0.36 e Å^−3^
Δρ_min_ = −0.68 e Å^−3^
Absolute structure: Flack (1983 ▶), 1428 Friedel pairs
Flack parameter: −0.03 (2)

### 

Data collection: *CrysAlis PRO* (Agilent Technologies, 2010 ▶); cell refinement: *CrysAlis PRO*; data reduction: *CrysAlis PRO*; program(s) used to solve structure: *SHELXS97* (Sheldrick, 2008 ▶); program(s) used to refine structure: *SHELXL97* (Sheldrick, 2008 ▶); molecular graphics: *X-SEED* (Barbour, 2001 ▶); software used to prepare material for publication: *publCIF* (Westrip, 2010 ▶).

## Supplementary Material

Crystal structure: contains datablocks global, I. DOI: 10.1107/S1600536811001930/si2326sup1.cif

Structure factors: contains datablocks I. DOI: 10.1107/S1600536811001930/si2326Isup2.hkl

Additional supplementary materials:  crystallographic information; 3D view; checkCIF report

## Figures and Tables

**Table 1:** Hydrogen-bond geometry (Å, °)

*D*—H⋯*A*	*D*—H	H⋯*A*	*D*⋯*A*	*D*—H⋯*A*
N2—H2⋯O2	0.88 (3)	1.89 (1)	2.745 (3)	166 (3)

**Fig. 1.**
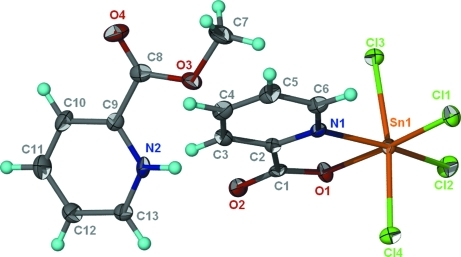
Thermal ellipsoid plot (Barbour, 2001) of [C7H8NO2]+ [SnCl4(C6H4NO2)]- at the 70% probability level; hydrogen atoms are drawn as spheres of arbitrary radius.

## supplementary crystallographic information

### Comment

The direct synthesis of a potentially chelating amino-carboxylic acid with stannic tetrachloride has not been reported. Pyridine-2-carboxylic acid yields a number of derivatives with organotin compounds; these are either synthesized by condensing the amino-carboxylic acids with an organotin oxide/hydroxide or by reacting the amino-carboxylic acids with an organotin chloride in the presence of a proton abstractor. With the latter route, the product may be an organostannate in which the pyridine-2-carboxylate chelates to the chlorine-bonded tin atom (Nowell *et al.*, 1983). In the reaction of pyridine-2-carboxylic acid and stannic chloride in methanol, one equivalent of the carboxylic acid is protonated at the amino site and is also esterified to yield the salt, [C_7_H_8_NO_2_]^+^ [SnCl_4_(C_6_H_4_NO_2_)]^-^ (Scheme I, Fig. 1). The tin atom in the anion is *N*,*O*-chelated by a pyridine-2-carboxylate in an octahedral geometry. The cation is linked to the anion by an N–H···O hydrogen bond (Table 1).

### Experimental

Stannic chloride pentahydrate 0.35 g, 1 mmol) and pyridine-2-carboxylic acid (0.13 g, 1 mmol) were loaded into a convection tube; the tube was filled with dry methanol and kept at 333 K. Colorless crystals were collected from the side arm after several days.

### Refinement

Carbon-bound H-atoms were placed in calculated positions [C—H 0.95 to 0.98 Å, *U*_iso_(H) 1.2 to 1.5*U*_eq_(C)] and were included in the refinement in the riding model approximation.

The amino H-atom was located in a difference Fourier map, and was refined with a distance restraint of N–H 0.88±0.01 Å; its temperature factor was refined.

### Crystal data

**Table:**

(C_7_H_8_NO_2_)[SnCl_4_(C_6_H_4_NO_2_)]	*F*(000) = 1016
*M**_r_* = 520.74	*D*_x_ = 1.870 Mg m^−^^3^
Orthorhombic, *P*2_1_2_1_2_1_	Mo *K*α radiation, λ = 0.71073 Å
Hall symbol: P 2ac 2ab	Cell parameters from 5063 reflections
*a* = 8.8898 (3) Å	θ = 2.5–29.3°
*b* = 10.3571 (3) Å	µ = 1.98 mm^−^^1^
*c* = 20.0938 (7) Å	*T* = 100 K
*V* = 1850.09 (10) Å^3^	Prism, colorless
*Z* = 4	0.30 × 0.25 × 0.20 mm

### Data collection

**Table:**

Agilent SuperNova Dual diffractometer with an Atlas detector	3787 independent reflections
Radiation source: SuperNova (Mo) X-ray Source	3679 reflections with *I* > 2σ(*I*)
Mirror	*R*_int_ = 0.021
Detector resolution: 10.4041 pixels mm^-1^	θ_max_ = 27.5°, θ_min_ = 2.5°
ω scans	*h* = −11→7
Absorption correction: multi-scan (*CrysAlis PRO*; Agilent Technologies, 2010)	*k* = −13→10
*T*_min_ = 0.588, *T*_max_ = 0.693	*l* = −25→15
6191 measured reflections	

### Refinement

**Table:**

Refinement on *F*^2^	Secondary atom site location: difference Fourier map
Least-squares matrix: full	Hydrogen site location: inferred from neighbouring sites
*R*[*F*^2^ > 2σ(*F*^2^)] = 0.021	H atoms treated by a mixture of independent and constrained refinement
*wR*(*F*^2^) = 0.049	*w* = 1/[σ^2^(*F*_o_^2^) + (0.0199*P*)^2^ + 1.6372*P*] where *P* = (*F*_o_^2^ + 2*F*_c_^2^)/3
*S* = 0.96	(Δ/σ)_max_ = 0.001
3787 reflections	Δρ_max_ = 0.36 e Å^−^^3^
222 parameters	Δρ_min_ = −0.68 e Å^−^^3^
1 restraint	Absolute structure: Flack (1983), 1428 Friedel pairs
Primary atom site location: structure-invariant direct methods	Flack parameter: −0.03 (2)

### Fractional atomic coordinates and isotropic or equivalent isotropic displacement parameters (Å^2^)

**Table:**

	*x*	*y*	*z*	*U*_iso_*/*U*_eq_	
Sn1	0.389388 (18)	0.500107 (17)	0.098352 (8)	0.01202 (5)	
Cl4	0.19677 (7)	0.49203 (9)	0.18312 (3)	0.01817 (13)	
Cl3	0.59780 (8)	0.48209 (7)	0.02047 (3)	0.01800 (14)	
Cl2	0.39844 (9)	0.72817 (6)	0.09456 (4)	0.02160 (15)	
Cl1	0.21001 (8)	0.46486 (7)	0.01310 (4)	0.02040 (16)	
O1	0.54531 (19)	0.5006 (2)	0.17598 (8)	0.0163 (4)	
O2	0.6707 (2)	0.3817 (2)	0.24920 (10)	0.0217 (5)	
O3	0.9194 (2)	0.2695 (2)	0.17094 (10)	0.0202 (4)	
O4	1.1105 (3)	0.1264 (2)	0.16977 (11)	0.0271 (5)	
N1	0.4309 (3)	0.2927 (2)	0.11697 (11)	0.0127 (5)	
N2	0.9440 (3)	0.3078 (2)	0.30014 (13)	0.0148 (5)	
H2	0.866 (2)	0.338 (3)	0.2787 (14)	0.012 (8)*	
C1	0.5869 (3)	0.3918 (3)	0.20127 (13)	0.0156 (6)	
C2	0.5262 (3)	0.2717 (3)	0.16754 (13)	0.0148 (6)	
C3	0.5707 (4)	0.1491 (3)	0.18569 (14)	0.0195 (6)	
H3	0.6361	0.1358	0.2225	0.023*	
C4	0.5172 (3)	0.0454 (3)	0.14858 (15)	0.0208 (6)	
H4	0.5467	−0.0402	0.1594	0.025*	
C5	0.4211 (3)	0.0678 (3)	0.09613 (15)	0.0196 (6)	
H5	0.3850	−0.0020	0.0700	0.024*	
C6	0.3776 (3)	0.1931 (3)	0.08186 (14)	0.0158 (6)	
H6	0.3088	0.2085	0.0466	0.019*	
C7	0.9024 (4)	0.2550 (3)	0.09935 (16)	0.0295 (7)	
H7A	0.8182	0.3084	0.0840	0.044*	
H7B	0.8824	0.1643	0.0887	0.044*	
H7C	0.9950	0.2827	0.0771	0.044*	
C8	1.0302 (4)	0.2023 (3)	0.19757 (16)	0.0182 (7)	
C9	1.0474 (3)	0.2317 (3)	0.27057 (14)	0.0154 (6)	
C10	1.1650 (4)	0.1834 (3)	0.30627 (15)	0.0209 (6)	
H10	1.2384	0.1302	0.2856	0.025*	
C11	1.1758 (4)	0.2136 (3)	0.37360 (17)	0.0266 (7)	
H11	1.2564	0.1803	0.3995	0.032*	
C12	1.0684 (4)	0.2923 (3)	0.40256 (17)	0.0239 (7)	
H12	1.0752	0.3138	0.4484	0.029*	
C13	0.9520 (4)	0.3391 (3)	0.36472 (15)	0.0177 (6)	
H13	0.8777	0.3932	0.3842	0.021*	

### Atomic displacement parameters (Å^2^)

**Table:**

	*U*^11^	*U*^22^	*U*^33^	*U*^12^	*U*^13^	*U*^23^
Sn1	0.01237 (8)	0.01123 (8)	0.01245 (8)	0.00035 (11)	−0.00144 (7)	−0.00058 (8)
Cl4	0.0168 (3)	0.0182 (3)	0.0195 (3)	−0.0011 (4)	0.0035 (2)	−0.0029 (3)
Cl3	0.0173 (3)	0.0198 (3)	0.0170 (3)	0.0010 (3)	0.0027 (3)	0.0005 (3)
Cl2	0.0263 (4)	0.0115 (3)	0.0270 (4)	0.0001 (3)	0.0020 (4)	−0.0006 (3)
Cl1	0.0186 (3)	0.0253 (4)	0.0173 (3)	0.0008 (3)	−0.0061 (3)	−0.0007 (3)
O1	0.0159 (8)	0.0182 (8)	0.0148 (8)	−0.0015 (12)	−0.0046 (7)	0.0011 (10)
O2	0.0170 (10)	0.0324 (11)	0.0156 (10)	0.0063 (10)	−0.0037 (9)	−0.0041 (9)
O3	0.0186 (11)	0.0297 (11)	0.0123 (9)	0.0005 (10)	−0.0002 (9)	−0.0031 (8)
O4	0.0294 (12)	0.0277 (10)	0.0242 (11)	0.0063 (12)	0.0070 (11)	−0.0067 (9)
N1	0.0132 (12)	0.0110 (10)	0.0139 (12)	0.0013 (10)	0.0015 (10)	0.0005 (8)
N2	0.0097 (12)	0.0163 (12)	0.0184 (13)	0.0012 (10)	−0.0026 (11)	0.0034 (10)
C1	0.0101 (13)	0.0242 (14)	0.0123 (12)	0.0042 (12)	0.0038 (11)	−0.0027 (11)
C2	0.0138 (13)	0.0190 (13)	0.0115 (13)	0.0018 (12)	0.0022 (11)	0.0021 (10)
C3	0.0210 (15)	0.0220 (14)	0.0155 (14)	0.0036 (13)	0.0025 (12)	0.0055 (11)
C4	0.0220 (15)	0.0150 (12)	0.0253 (16)	0.0023 (12)	0.0100 (13)	0.0063 (11)
C5	0.0212 (14)	0.0132 (12)	0.0245 (14)	−0.0020 (12)	0.0076 (14)	−0.0009 (11)
C6	0.0153 (13)	0.0151 (12)	0.0170 (14)	−0.0025 (12)	0.0034 (12)	−0.0015 (10)
C7	0.0268 (17)	0.0482 (19)	0.0134 (14)	0.0025 (18)	−0.0033 (17)	−0.0023 (15)
C8	0.0177 (15)	0.0183 (14)	0.0186 (16)	−0.0025 (13)	0.0012 (14)	−0.0014 (12)
C9	0.0145 (13)	0.0136 (12)	0.0181 (14)	−0.0013 (11)	0.0008 (12)	0.0009 (10)
C10	0.0172 (14)	0.0204 (14)	0.0253 (16)	0.0060 (12)	−0.0009 (14)	−0.0009 (12)
C11	0.0224 (16)	0.0333 (17)	0.0241 (16)	0.0063 (15)	−0.0067 (15)	0.0012 (14)
C12	0.0242 (16)	0.0292 (15)	0.0183 (14)	0.0027 (14)	−0.0047 (15)	−0.0018 (14)
C13	0.0184 (14)	0.0180 (13)	0.0165 (14)	0.0019 (12)	−0.0012 (12)	−0.0015 (11)

### Geometric parameters (Å, °)

**Table:**

Sn1—O1	2.0868 (16)	C3—H3	0.9500
Sn1—N1	2.211 (2)	C4—C5	1.376 (4)
Sn1—Cl2	2.3647 (6)	C4—H4	0.9500
Sn1—Cl1	2.3687 (7)	C5—C6	1.384 (4)
Sn1—Cl4	2.4167 (6)	C5—H5	0.9500
Sn1—Cl3	2.4325 (7)	C6—H6	0.9500
O1—C1	1.291 (3)	C7—H7A	0.9800
O2—C1	1.222 (3)	C7—H7B	0.9800
O3—C8	1.320 (4)	C7—H7C	0.9800
O3—C7	1.454 (4)	C8—C9	1.506 (4)
O4—C8	1.199 (4)	C9—C10	1.363 (4)
N1—C2	1.341 (4)	C10—C11	1.392 (4)
N1—C6	1.337 (3)	C10—H10	0.9500
N2—C13	1.339 (4)	C11—C12	1.384 (5)
N2—C9	1.348 (4)	C11—H11	0.9500
N2—H2	0.88 (2)	C12—C13	1.373 (4)
C1—C2	1.516 (4)	C12—H12	0.9500
C2—C3	1.379 (4)	C13—H13	0.9500
C3—C4	1.391 (4)		
			
O1—Sn1—N1	76.42 (9)	C5—C4—H4	120.2
O1—Sn1—Cl2	89.93 (7)	C3—C4—H4	120.2
N1—Sn1—Cl2	165.94 (7)	C4—C5—C6	119.3 (3)
O1—Sn1—Cl1	171.19 (7)	C4—C5—H5	120.3
N1—Sn1—Cl1	94.88 (6)	C6—C5—H5	120.3
Cl2—Sn1—Cl1	98.83 (3)	N1—C6—C5	121.0 (3)
O1—Sn1—Cl4	86.78 (5)	N1—C6—H6	119.5
N1—Sn1—Cl4	88.02 (6)	C5—C6—H6	119.5
Cl2—Sn1—Cl4	94.67 (3)	O3—C7—H7A	109.5
Cl1—Sn1—Cl4	91.57 (2)	O3—C7—H7B	109.5
O1—Sn1—Cl3	88.58 (5)	H7A—C7—H7B	109.5
N1—Sn1—Cl3	84.67 (6)	O3—C7—H7C	109.5
Cl2—Sn1—Cl3	91.72 (3)	H7A—C7—H7C	109.5
Cl1—Sn1—Cl3	92.04 (2)	H7B—C7—H7C	109.5
Cl4—Sn1—Cl3	172.10 (3)	O4—C8—O3	126.9 (3)
C1—O1—Sn1	118.83 (19)	O4—C8—C9	121.7 (3)
C8—O3—C7	115.1 (2)	O3—C8—C9	111.3 (3)
C2—N1—C6	119.9 (2)	N2—C9—C10	120.3 (3)
C2—N1—Sn1	113.08 (18)	N2—C9—C8	118.6 (3)
C6—N1—Sn1	126.95 (19)	C10—C9—C8	121.1 (3)
C13—N2—C9	122.2 (3)	C9—C10—C11	118.8 (3)
C13—N2—H2	116 (2)	C9—C10—H10	120.6
C9—N2—H2	122 (2)	C11—C10—H10	120.6
O2—C1—O1	124.0 (3)	C10—C11—C12	119.6 (3)
O2—C1—C2	119.9 (2)	C10—C11—H11	120.2
O1—C1—C2	116.0 (2)	C12—C11—H11	120.2
N1—C2—C3	122.1 (3)	C13—C12—C11	119.7 (3)
N1—C2—C1	115.5 (2)	C13—C12—H12	120.1
C3—C2—C1	122.4 (3)	C11—C12—H12	120.1
C2—C3—C4	118.1 (3)	N2—C13—C12	119.4 (3)
C2—C3—H3	121.0	N2—C13—H13	120.3
C4—C3—H3	121.0	C12—C13—H13	120.3
C5—C4—C3	119.5 (3)		
			
N1—Sn1—O1—C1	−3.82 (19)	O1—C1—C2—C3	174.6 (3)
Cl2—Sn1—O1—C1	179.60 (18)	N1—C2—C3—C4	1.8 (4)
Cl4—Sn1—O1—C1	84.92 (18)	C1—C2—C3—C4	−175.4 (3)
Cl3—Sn1—O1—C1	−88.68 (18)	C2—C3—C4—C5	−0.9 (4)
O1—Sn1—N1—C2	2.07 (18)	C3—C4—C5—C6	−1.0 (4)
Cl2—Sn1—N1—C2	16.3 (4)	C2—N1—C6—C5	−1.1 (4)
Cl1—Sn1—N1—C2	−176.51 (18)	Sn1—N1—C6—C5	175.4 (2)
Cl4—Sn1—N1—C2	−85.10 (19)	C4—C5—C6—N1	2.0 (4)
Cl3—Sn1—N1—C2	91.89 (19)	C7—O3—C8—O4	−4.7 (5)
O1—Sn1—N1—C6	−174.6 (3)	C7—O3—C8—C9	175.2 (3)
Cl2—Sn1—N1—C6	−160.4 (2)	C13—N2—C9—C10	0.1 (4)
Cl1—Sn1—N1—C6	6.8 (2)	C13—N2—C9—C8	−179.6 (2)
Cl4—Sn1—N1—C6	98.3 (2)	O4—C8—C9—N2	−173.4 (3)
Cl3—Sn1—N1—C6	−84.8 (2)	O3—C8—C9—N2	6.7 (4)
Sn1—O1—C1—O2	−175.6 (2)	O4—C8—C9—C10	7.0 (5)
Sn1—O1—C1—C2	4.8 (3)	O3—C8—C9—C10	−173.0 (3)
C6—N1—C2—C3	−0.9 (4)	N2—C9—C10—C11	0.4 (4)
Sn1—N1—C2—C3	−177.8 (2)	C8—C9—C10—C11	180.0 (3)
C6—N1—C2—C1	176.5 (2)	C9—C10—C11—C12	−0.6 (5)
Sn1—N1—C2—C1	−0.4 (3)	C10—C11—C12—C13	0.4 (5)
O2—C1—C2—N1	177.7 (2)	C9—N2—C13—C12	−0.3 (4)
O1—C1—C2—N1	−2.7 (4)	C11—C12—C13—N2	0.0 (5)
O2—C1—C2—C3	−5.0 (4)		

### Hydrogen-bond geometry (Å, °)

**Table:**

*D*—H···*A*	*D*—H	H···*A*	*D*···*A*	*D*—H···*A*
N2—H2···O2	0.88 (3)	1.89 (1)	2.745 (3)	166 (3)

## References

[bb1] Agilent Technologies (2010). *CrysAlis PRO* Agilent Technologies, Yarnton, England.

[bb2] Barbour, L. J. (2001). *J. Supramol. Chem.* **1**, 189–191.

[bb3] Flack, H. D. (1983). *Acta Cryst.* A**39**, 876–881.

[bb4] Nowell, I. W., Brooks, J. S., Beech, G. & Hill, R. (1983). *J. Organomet. Chem.* **244**, 119–124.

[bb5] Sheldrick, G. M. (2008). *Acta Cryst.* A**64**, 112–122. 10.1107/S0108767307043930 18156677

[bb6] Westrip, S. P. (2010). *J. Appl. Cryst.* **43**, 920–925.

